# Supplementary material for: Content-rich biological network constructed by mining PubMed abstracts
Source: BMC Bioinformatics. 2004 Oct 8;5:147. doi: 10.1186/1471-2105-5-147 (PMC528731; doi:10.1186/1471-2105-5-147)
Supplement: Additional File 2 — The original results of the above study (non-essential files are deleted to keep the file size under the limit set by BMC bioinformatics). [file 1471-2105-5-147-S2.bz2 › chilibotAdditionalFile2/dip10/679ID9514722E11088/html/MAUB_MAUA.html]

 


 **MAUB** and **MAUA** 
  
Found 74 abstracts in PubMed, retrieved 10.  
 

 What does Google say? 
 PDF only 
| .edu only 

---

**Interactive relationship** (e.g. stimulation, inhibition, etc)

**Neutral relationship**- This alphaF55A MADH  [ **MAUA** ]   [ **MAUB** ]  exhibits a 400 fold lower Km value for histamine than does native MADH  [ **MAUA** ]   [ **MAUB** ]  when assayed in solution.  Ref: 11924976 Anal Chem, 2002
- For each mutant, the majority of the protein that was isolated was inactive and exhibited weaker subunit subunit interactions than native MADH  [ **MAUA** ]   [ **MAUB** ] .  Ref: 12641453 Biochemistry, 2003
- The biogenesis of the quinone cofactor tryptophan tryptophylquinone TTQ in methylamine dehydrogenase MADH  [ **MAUA** ]   [ **MAUB** ]  involves the post translational modification of two constitutive Trp residues Trp beta 57 and Trp beta 108 in Paracoccus denitrificans MADH  [ **MAUA** ]   [ **MAUB** ] .  Ref: 12641453 Biochemistry, 2003
- The alphaF55A MADH  [ **MAUA** ]   [ **MAUB** ]  immobilized on the electrode exhibited Michaelis Menten behavior in response to varied concentrations of histamine with an approximately 3 fold lower Km value than that exhibited by the immobilized native MADH  [ **MAUA** ]   [ **MAUB** ] .  Ref: 11924976 Anal Chem, 2002
- alphaF55A MADH  [ **MAUA** ]   [ **MAUB** ]  exhibits an increased K d for cation dependent spectral changes and a decreased K d for cation dependent stimulation of the rate of gated ET from N quinol MADH  [ **MAUA** ]   [ **MAUB** ]  to amicyanin.  Ref: 12686138 Biochim Biophys Acta, 2003
- The detection limit for the native MADH  [ **MAUA** ]   [ **MAUB** ]  PPy sensor was approximately 20 microM while the alphaF55A MADH  [ **MAUA** ]   [ **MAUB** ]  PPy sensor exhibited a detection limit of approximately 5 microM, a 4 fold increase compared to the native MADH  [ **MAUA** ]   [ **MAUB** ]  PPy sensor.  Ref: 11924976 Anal Chem, 2002

**Non-interactive relationship** (e.g. studied together, co-existance, homology, etc.)

- To further modulate the substrate specificity of MADH  [ **MAUA** ]   [ **MAUB** ] , the side chain of alphaPhe 55 was repositioned by site directed mutagenesis of residue betaIle 107.  Ref: 11733518 J Biol Chem, 2002
- In contrast, with alphaF55E MADH  [ **MAUA** ]   [ **MAUB** ]  cross linking of subunits is observed.  Ref: 12062431 FEBS Lett, 2002
- Thus, it has been possible to create forms of MADH  [ **MAUA** ]   [ **MAUB** ]  that exhibit a preference for amines with carbon chain lengths of one, three, five, or seven carbons.  Ref: 11733518 J Biol Chem, 2002
- Using site directed mutagenesis, phenylalanine 55 on the alpha subunit of MADH  [ **MAUA** ]   [ **MAUB** ]  was converted to alanine.  Ref: 11924976 Anal Chem, 2002
- Site directed mutagenesis of either alphaPhe 55 or betaIle 107 a residue in the beta subunit which interacts with alphaPhe 55 converts MADH  [ **MAUA** ]   [ **MAUB** ]  into enzymes with specificities for long chain amines, amylamine or propylamine.  Ref: 12686138 Biochim Biophys Acta, 2003
- Methylamine dehydrogenase MADH  [ **MAUA** ]   [ **MAUB** ]  may be immobilized in a polypyrrole PPy film on an electrode surface and used as an amperometric sensor for the determination of histamine.  Ref: 11924976 Anal Chem, 2002
- Paracoccus denitrificans produces two primary enzymes for the amine oxidation, tryptophan tryptophylquinone TTQ containing methylamine dehydrogenase MADH  [ **MAUA** ]   [ **MAUB** ]  and quinohemoprotein amine dehydrogenase QH AmDH.  Ref: 12686147 Biochim Biophys Acta, 2003
- Methylamine dehydrogenase MADH  [ **MAUA** ]   [ **MAUB** ]  is a tryptophan tryptophylquinone dependent enzyme that catalyzes the oxidative deamination of primary amines.  Ref: 11733518 J Biol Chem, 2002
- The resulting inactivation is accompanied by the formation of a covalent cross link between the alpha and beta subunits of MADH  [ **MAUA** ]   [ **MAUB** ] .  Ref: 12062431 FEBS Lett, 2002
- These properties are in marked contrast with those of MADH  [ **MAUA** ]   [ **MAUB** ] , which shows high specificity to amicyanin.  Ref: 12686147 Biochim Biophys Acta, 2003
- Methylamine dehydrogenase MADH  [ **MAUA** ]   [ **MAUB** ]  possesses an alpha 2 beta 2 structure with each smaller beta subunit possessing a tryptophan tryptophylquinone TTQ prosthetic group.  Ref: 12437349 Biochemistry, 2002
- Native MADH  [ **MAUA** ]   [ **MAUB** ]  exhibits a strong preference for methylamine over longer carbon chain amines.  Ref: 11733518 J Biol Chem, 2002
- The crystal structure of alphaF55A MADH  [ **MAUA** ]   [ **MAUB** ]  has also been determined with phenylhydrazine covalently bound to TTQ in the active site.  Ref: 12437349 Biochemistry, 2002
- Direct dynamics calculations employing hybrid quantum mechanical and molecular mehanical QM MM potentials and molecular dynamics simulation methods have been used to explore the important dynamic role that enzyme structure has on proton transfer in the C H bond breakage of a methylamine substrate by methylamine dehydrogenase MADH  [ **MAUA** ]   [ **MAUB** ] .  Ref: 12555860 Faraday Discuss, 2003
- Methylamine dehydrogenase MADH  [ **MAUA** ]   [ **MAUB** ]  possesses an alpha 2 beta 2 subunit structure with each smaller beta subunit possessing a tryptophan tryptophylquinone TTQ prosthetic group.  Ref: 12686138 Biochim Biophys Acta, 2003
- It is now shown that the alphaF55A mutation also increases the rate of the true electron transfer ET reaction from O quinol MADH  [ **MAUA** ]   [ **MAUB** ]  to amicyanin.  Ref: 12437349 Biochemistry, 2002
- The biosynthesis of methylamine dehydrogenase MADH  [ **MAUA** ]   [ **MAUB** ]  from Paracoccus denitrificans requires four genes in addition to those that encode the two structural protein subunits.  Ref: 12809487 Biochemistry, 2003
- One of these, mauG, exhibits sequence similarity to diheme cytochrome c peroxidases and is required for the synthesis of the tryptophan tryptophylquinone TTQ prosthetic group of MADH  [ **MAUA** ]   [ **MAUB** ] .  Ref: 12809487 Biochemistry, 2003
- The crystal structure of alphaF55A MADH  [ **MAUA** ]   [ **MAUB** ]  in complex with its electron acceptors, amicyanin and cytochrome c 551i, has been determined.  Ref: 12437349 Biochemistry, 2002
- No cross linking is seen with alphaF55A or alphaF55I MADH  [ **MAUA** ]   [ **MAUB** ]  mutants.  Ref: 12062431 FEBS Lett, 2002
- betaI107V MADH  [ **MAUA** ]   [ **MAUB** ]  exhibits a strong preference for propylamine, and betaI107N MADH  [ **MAUA** ]   [ **MAUB** ]  exhibits a preference for 1 aminopentane.  Ref: 11733518 J Biol Chem, 2002
- Methylamine dehydrogenase MADH  [ **MAUA** ]   [ **MAUB** ]  is produced by a range of gram negative methylotrophic and autotrophic bacteria, and allows the organisms to utilise methylamine as the sole source of carbon.  Ref: 12686162 Biochim Biophys Acta, 2003
- A stable binary MADH  [ **MAUA** ]   [ **MAUB** ]  amicyanin complex can be formed, and its crystal structure has been solved to 2.5 A resolution by Chen et al.  Ref: 12686162 Biochim Biophys Acta, 2003
- Site directed mutagenesis studies have revealed several roles for this residue in catalysis and electron transfer ET by MADH  [ **MAUA** ]   [ **MAUB** ] .  Ref: 12686138 Biochim Biophys Acta, 2003
- An alphaF55A MADH  [ **MAUA** ]   [ **MAUB** ]  PPy sensor was constructed, and its properties were compared to that of the native MADH  [ **MAUA** ]   [ **MAUB** ]  PPy sensor.  Ref: 11924976 Anal Chem, 2002
- Cyclopropylamine is a mechanism based inhibitor of the quinoprotein methylamine dehydrogenase MADH  [ **MAUA** ]   [ **MAUB** ]  from Paracoccus denitrificans.  Ref: 12062431 FEBS Lett, 2002
- When alphaPhe 55 is replaced with Ala, this preference is reversed with alphaF55A MADH  [ **MAUA** ]   [ **MAUB** ]  preferring long chain amines with at least seven carbons Zhu, Z.  Ref: 11733518 J Biol Chem, 2002
- To complete the catalytic cycle, MADH  [ **MAUA** ]   [ **MAUB** ]  is reoxidised via an electron transfer ET chain.  Ref: 12686162 Biochim Biophys Acta, 2003
